# Editorial: Rising stars in nutrition and microbes: nutrients and diets that shape gut microbiota, change intestinal barrier and regulate lifestyle-related diseases

**DOI:** 10.3389/fnut.2023.1328660

**Published:** 2023-11-20

**Authors:** Peng-Gao Li, Zhen-Wu Huang

**Affiliations:** ^1^School of Public Health, Capital Medical University, Beijing, China; ^2^Beijing Key Laboratory of Environmental Toxicology, Beijing, China; ^3^Beijing Key Laboratory of Clinical Epidemiology, Beijing, China; ^4^National Institute for Nutrition and Health, Chinese Center for Disease Control and Prevention, Beijing, China

**Keywords:** nutrients, diets, gut microbiota, intestinal barrier, lifestyle-related diseases

The digestion of food and the absorption of nutrients and other bioactive substances are mainly completed in the stomach and small intestine. The remaining food residue will enter the large intestine and be further decomposed by the microbial communities residing there. This procedure will release more nutrients from food and produce new products that may be beneficial or harmful to the host, and have a significant impact on the host's gut and overall health. Therefore, the intestinal microbiota is now considered an integral part of the body that plays an important role in the development of many local and systemic diseases, such as inflammatory bowel disease, colorectal cancer, obesity, type 2 diabetes, and Alzheimer's disease (1).

In recent years, many scientists have shifted their interest to the interaction between diet, gut microbiota, and host health, and proposed to prevent diseases by regulating the composition of gut microbiota. In many experimental and clinical studies, dietary approaches to prevent diseases, such as adding probiotics, prebiotics, or synbiotics to food, have been proven to be safe and effective. The mechanisms by which specific foods or food components regulate specific bacterial species in the gut, the genes responsible for bacterial activity, the bioactive products they produce, and the pathophysiological effects of these products in specific bodily organs are increasingly being recognized. However, there are still many knowledge gaps in this field. For example, if substances from food sources can be absorbed without the help of the gut microbiota, it is difficult to distinguish between their direct effects and those mediated by the so-called “gut-X axis.” It is also very difficult to elucidate the exact mechanism(s) by which gut bacteria mediate the impact of food components on host pathophysiological responses even with the help of so many “-omics” technologies.

To add insights to this field, in this e-book, the role of diet, food, or food components in shaping the gut microbiota and preventing lifestyle-related diseases was studied in various experimental models, with a focus on changes in gut barrier function and the inflammatory reactions induced during this process. Kadyan et al. found that resistant starch (RS) extracted from four different dietary pulse varieties can suppress aging-related metabolic disorders by regulating the gut microbiome of mice and ameliorating high-fat diet-induced gut leakiness and inflammation. Interestingly, they find that the impact of RS is food source- and gender-specific, and called for deeper research on the complexity of RS from aspects such as botanical origin, microstructure, impact on the gut microbiome, and the resulting pathophysiological responses of different genders (Kadyan et al.). Li et al. show that the fermentation of Chinese medicinal food *Astragalus membranaceus* (FA) has produced many new bioactive chemicals, significantly enhancing its preventive effect on dextran sulfate sodium (DSS)-induced colitis and dysbiosis in mice. The correlation between key gut microbes that produce short-chain fatty acids (SCFA), such as *Akkermansia* and *Alistipes*, and colitis parameters explains the role of FA in balancing the immune responses and repairing damaged intestinal mucosa (Li et al.). The authors also acknowledged that it is difficult to distinguish between the effects of FA and those of FA-affected gut microbiota, which is a common problem faced by research aimed at establishing the mediating role of gut microbiota in response to oral substances, as they cannot rule out the direct effect of FA absorption into the body and exerting a systemic effect before encountering the gut microbiota. Zhang et al. observed the preventive effect of supplementing two types of algal oil on antibiotic-induced opportunistic pathogen infection and inflammation. They indicate that *Parabacteroides* and *Melissococus* were the main culprits of gut leakiness and inflammation caused by ceftriaxone sodium (CS) while supplementing with algal oil increased the abundance of a cohort of beneficial bacteria, reduced the bad ones, and repressed CS-induced inflammatory damages. They further analyzed the correlation between changes in gut microbiota and the fatty acid composition of algal oil and revealed interesting results (Zhang et al.).

To prevent lifestyle-related chronic diseases in adulthood, it has been proposed to use infant formula (IF) containing probiotics and prebiotics to help newborns establish a healthy gut microbiota. Therefore, Bakshi et al. reviewed the formulation and production of IF and discussed its impact on the establishment of infant gut microbiota and its short- and long-term health consequences.

In summary, in a world where adding bioactive ingredients to food products to nurture a robust and balanced gut microbiota and promote consumer health is increasingly becoming a trend, we should invest more in this research field to generate more practical insights to elevate our understanding of this hot topic and help us find better-targeted, personalized approaches for improving health, delaying aging and reducing the burden of disease.

## Author contributions

P-GL: Writing—original draft, Writing—review & editing. Z-WH: Writing—review & editing.
